# The role of microbiota and toll-like receptors in polycystic ovary syndrome: regulatory mechanisms of androgen metabolism

**DOI:** 10.1530/EC-25-0162

**Published:** 2025-06-21

**Authors:** Xueming Huang, Liuyan Wu, Yunxiang Zhang, Xiaowen Lai, Dan Sun

**Affiliations:** Guangxi Medical University, Nanning, Guangxi, China

**Keywords:** polycystic ovary syndrome (PCOS), microbiota, androgen, toll-like receptors (TLRs)

## Abstract

Polycystic ovary syndrome (PCOS), a prevalent reproductive endocrine and metabolic disorder in gynecology, has hyperandrogenism (HA) as an essential pathophysiological alteration. PCOS patients, with or without HA, present diverse clinical manifestations. The function of intestinal or reproductive tract microorganisms in PCOS has drawn attention in recent years and is associated with the occurrence of HA. In this article, we review the connection between the microbial alterations in the intestinal and reproductive tracts and androgens in PCOS, and elaborate on the role of TLRs in this process.

## Background

Polycystic ovary syndrome (PCOS) is a common reproductive endocrine disease combined with metabolic disorders. In 1935, Stein and Leventhal first reported cases of amenorrhea and polycystic ovary as the main manifestations combined with hirsutism and obesity (1). Therefore, PCOS is also known as Stein-Leventhal syndrome (2). The global incidence of PCOS is 6–21% (3), and women of childbearing age account for 8–13% (4). The main clinical features of the disease are oligoovulation or anovulation (OA), biochemical or clinical manifestations of hyperandrogen (HA), and polycystic ovarian changes (PCO). It can cause infertility and metabolic diseases such as obesity, insulin resistance, type 2 diabetes, and cardiovascular disease (5). Currently, the Rotterdam standard is widely adopted internationally, according to which PCOS can be divided into four types: type A, OA + HA + PCO; type B, OA + HA; type C, HA + PCO; and type D, OA + PCO (5). PCOS with HA is more common, but some clinical symptoms of PCOS without HA are lighter than those in the presence of HA. Compared with types A and B, type D has the lightest menstrual disorder symptoms (3). Moreover, type D has the mildest biochemical changes among the four types (6), and the incidence and degree of insulin resistance, glucose and lipid metabolism disorders, and metabolic syndrome are lower than those of the other three types (6, 7, 8). Therefore, we hypothesized that hyperandrogen may play a special role in the formation of clinical phenotypes and biochemical metabolic differences in PCOS patients.

Intestinal microecology is an important microecosystem of the human body and is considered to be the second-largest endocrine organ (9, 10). The gut microbiota constitutes a biological barrier of the body, which plays a key role in the processes of nutrition metabolism, sex hormone regulation, immune barrier development and maturity, and resistance to harmful microorganisms (11, 12, 13). Breaking the homeostasis between the gut microbiota and the body can cause a series of diseases such as metabolic syndrome, obesity, type 2 diabetes, inflammatory bowel disease, and cancer (9, 14). The microecology of the lower reproductive tract is simpler. With hydrogen peroxide-producing *Lactobacillus* as the dominant flora, a natural barrier is formed on the vaginal mucosal surface to maintain the stability of the vaginal microecology (15). The regulation of vaginal microbiota is closely related to changes in sex hormones (16). Studies have found that the vaginal microbiota of PCOS patients is different from that of the normal population (17). In addition, the vaginal microbiome is associated with specific clinical manifestations of PCOS, including acanthosis nigricans, intermenstrual bleeding, pregnancy history, testosterone levels, and anti-Müllerian hormone levels. The abundance of *L. crispatus* is higher, while that of *L. iners* is lower among PCOS patients with elevated testosterone levels (18).

In this review, we briefly introduce the structure, function, source, and transformation of androgens, as well as toll-like receptors (TLRs) and TLR pathways. Furthermore, we focus on reviewing the relationship between TLRs and the gut microbiota and lower reproductive tract microbiota of PCOS. It is of great significance to study how to change the microbiota to reduce peripheral blood androgen levels in PCOS patients, thereby improving the clinical phenotype and metabolism.

## Androgens

Androgens in the human body mainly include dehydroepiandrosterone sulfate (DHEA-S), dehydroepiandrosterone (DHEA), androstenedione, testosterone, and dihydrotestosterone (DHT), among which androstenedione and DHEA are precursors of androgens (19). Androgens are some of the most important hormones. The average serum testosterone level of women of childbearing age is 7 nM (20). Androgens in women can be derived from the ovary and adrenal cortex, while hyperandrogen in PCOS patients mainly originates from the ovary (21). The main androgens produced by the ovary are androstenedione and testosterone. Androstenedione is mainly synthesized and secreted by follicular membrane cells, and testosterone is mainly synthesized and secreted by ovarian stromal cells and portal cells (22). Cholesterol is the precursor for the synthesis of androgens. Under the sequential action of rapid regulator protein, cholesterol side chain lyase, 17α-hydroxylase, 17,20-lyase, 3β-hydroxysteroid dehydrogenase, and 17β-hydroxysteroid dehydrogenase, androgens are synthesized or transformed in the adrenal cortex, amphoteric gonads, and peripheral tissues (22). Cytochrome P450, hydroxysteroid dehydrogenase, oxidoreductase, sulfotransferase, UDP-glucosyltransferase (UGT), and other enzymes regulate androgen homeostasis in the human body (23, 24). UDP-glucuronic acid glycosyltransferase is a key enzyme involved in androgen hepatoenteric circulation. UGT converts androgens into glucuronic acid conjugates in the liver and intestines so that androgens can be excreted through the urine (25). In addition, under the action of 5-reductase type 1 enzyme (SRD5a1), 5-reductase type 2 enzyme (SRD5a2), and 5-reductase type 3 enzyme (SRD5a3), androgens can transition between active and inactive states. Studies have found that in certain male reproductive tissues, such as the prostate and seminal vesicles, testosterone is converted into the more potent androgen DHT by SRD5a2, whereas in the liver, mainly SRD5a1 converts testosterone into DHT (26). SRD5a3 has been identified (27), but its function remains unclear (28).

Androgens are essential for both reproductive and non-reproductive organs (29). Androgens can regulate follicular development (30), improve female sexual desire and sexual dysfunction (31, 32). Topical use of androgens can relieve the symptoms of vaginal dryness and vaginal atrophy (33, 34, 35). Studies have found that the levels of endogenous androgens are inversely proportional to the risk of cardiovascular disease (36, 37, 38, 39). Androgens also have a protective effect on the nervous system (40, 41). Low androgen levels after menopause reduce cognitive ability and increase the risk of dementia (42, 43). Androgens are important in bone and muscle synthesis; they can stimulate erythropoiesis and promote the reabsorption of water and sodium by distal convoluted tubules (19). However, in women, excessive androgens can lead to signs of hyperandrogenism, such as hirsutism and acne, cardiovascular disease, and type 2 diabetes, and promote the accumulation of body fat (44, 45), which can cause menstrual disorders and follicular atresia (46, 47). Hyperandrogenemia is an important manifestation of PCOS.

## Androgens interact with gut microbiota

Androgens have certain effects on the gut microbiota, which vary with the gender of mammalian hosts. Mueller found that healthy male subjects had a higher Bacteroides-Prevotella abundance than females, while there was no difference between men and postmenopausal women (48). The diversity of gut microbiota in adolescent males is less than that in females, and the microbiota contributes to increased levels of testosterone in the blood (49). Compared with females, the abundance of *Ruminococcus*, *Coprococcus*, and *Dorea* in healthy male mice is much higher (50, 51). The changes of hormones and gut microbiota in female and male recipients were detected using transgender fecal microbiota transplantation (FMT). Compared with male mice that received same-sex FMT, the testosterone concentration in cross-sex male recipients was significantly reduced, and several bacterial taxa were correlated with plasma testosterone levels (52). The research by Wu *et al.* found that the relative abundances of *Muribaculaceae*, *Turicibacter*, and *Parasutterella* in the female intestinal mucosa group of mice were significantly higher than those in the male intestinal mucosa group. Among them, *Turicibacter* and *Parasutterella* were negatively correlated with testosterone (53). Furthermore, a study using single and multiple linear regression analyses demonstrated the correlation between hyperandrogenism and microbiota diversity. The results indicated that hyperandrogenemia might play a key role in altering the gut microbiota in women with PCOS (54). Meanwhile, it has been suggested that sex steroids are a carbon source for intestinal bacteria, which participate in the metabolism of sexual sterols (55). Moadi’s study found that testosterone affects the microbiota of fecal samples collected before puberty in mice and that testosterone supplementation *in vivo* affects the gut microbiota of both male and female mice (52).

There is evidence that gut microbiota can regulate local and systemic androgen levels. Like estrogen, androgen is incorporated in the liver and excreted into the intestine through the bile duct tree (25). Research has found that the gut microbiota is involved in intestinal metabolism and can deglucuronidate DHT and testosterone, which leads to extremely high levels of DHT in the male colon (13). A prospective study, which was designed to assess the effects of urine contamination by selected pathogens on the endogenous androgenic steroid profile, has found that *Escherichia coli* can uncouple androgens into an active free state (56). Li *et al.* isolated *Mycobacterium neoaurum* from stool samples of testosterone-deficient patients with depression and showed that this strain can degrade testosterone *in vitro* (57). Androgen-uncoupling enzymes can also be produced by the gut microbiome, which increases circulating androgen levels (13, 58).

The microbiome can also increase local androgen levels through another mechanism. There is evidence that *Escherichia coli* and *Bacteroides* can synthesize androgens from bile acids (59, 60). *Clostridium scindens*, which is involved in bile acid biotransformation, has also been shown to convert glucocorticoids into androgens (61). These studies provide a mechanism by which the microbiome can directly change the exposure of the intestinal epithelium to sex steroids.

## Gut microbiota may be responsible for hyperandrogenemia in PCOS

The mechanism of hyperandrogen in PCOS is still unclear, but the mainstream view is that androgens are mainly secreted from the ovary (21), and excessive secretion of androgens by the ovary is related to gut microbiota disorders. It has been reported that α diversity is decreased in PCOS patients of different ages (54, 62, 63, 64, 65), and β diversity is also changed (54, 62, 66). The relative abundance of microbiota was different. It has been found that *Bifidobacterium* and *Lactobacillus* are negatively correlated with PCOS (67, 68, 69), while *Bacteroides*, *Prevotella*, *Blautia*, and *Roseburia* are positively correlated with PCOS (67, 68, 69, 70, 71). A study showed that differences in gut microbiota between PCOS patients and controls differ in observational studies. Compared with controls, the gut microbiota taxa associated with increased risk in PCOS patients were *Streptococcus*, *Streptococcaceae*, *Ruminococcus*, and *Actinomyces* (72). In 2012, Tremellen & Pearce initially proposed that the disturbance of gut microbiota can increase the permeability of the intestinal mucosa in PCOS patients and promote the entry of lipopolysaccharides into systemic circulation, thus activating the immune system and interfering with insulin receptor function, increasing serum insulin levels, promoting androgen secretion by the ovaries, and affecting normal follicular development (73). Furthermore, it has also been found that the lower diversity of gut microbiota in PCOS patients, the destruction of the intestinal barrier, and the production of endotoxin may be related to the clinical phenotype of PCOS (74).

Recently, however, there have been studies suggesting that intestinal microecological disorder is another cause of hyperandrogenism in PCOS patients or animal models. In both human and animal studies, it has been found that *Bacteroides*, *Streptococcus*, and *Prevotella* are positively correlated with high androgen levels in PCOS (54, 66). To study the influence of gut microbiota on PCOS, Qi *et al.* transferred the feces of healthy females and PCOS patients into mice. The results showed that compared with the control group, PCOS-transplanted mice showed insulin resistance and a disordered estrus cycle. In addition, the number of cystoid follicles increased, and corpus luteum decreased in PCOS-transplanted mice. Compared with the control group, the number of first litters of PCOS-transplanted mice decreased after mating. The results of genome-wide shotgun sequencing showed that the abundance of *B. vulgatus* in PCOS patients increased significantly. Gavage of *B. vulgatus* to wild-type recipient mice showed that *B. vulgatus* could induce insulin resistance in mice, estrus cycle disruption, changes in ovarian morphology, and hormone levels (75). Lactic acid bacteria can affect testosterone levels by regulating intestinal flora such as *Akkermansia*, *Roseburia*, *Prevotella*, *Staphylococcus,* and *Lactobacillus*, so as to alleviate the hyperandrogenic symptoms of PCOS (76). In addition, Wong’s study found that *B. vulgatus* promoted PCOS by inhibiting glucagon-like peptide-1 (GLP-1), which increased the release of androgen androstenedione from muscle (77).

In addition, the hyperandrogens influences the gut microbiota to some extent. Researchers used letrozole to induce hyperandrogenism in a mouse model and found that the gut microbiota changed significantly. The longer the administration of letrozole, the more significant the changes in the gut microbiota become. It is hypothesized that hyperandrogenism may significantly change the gut microbiota, and this effect is independent of diet (67). This study suggests that the gut microbiota imbalance is caused by HA instead of being the direct effect of letrozole. In addition, it has recently been found that alpha diversity and beta diversity of gut microbiota are related to HA, which indicates that hyperandrogenism in PCOS is related to changes in gut microbiome composition (54, 66). Using DHEA and a high-fat diet to generate a mouse PCOS model, we found that HA changed the β-diversity of the gut microbiome (78).

In summary, we hypothesize that the gut microbiota is another endocrine organ that produces hyperandrogenism in patients with PCOS. Adjusting gut microecology through microbiota transplantation may be a new approach for the treatment of PCOS.

## The role of TLRs and TLR signaling in the intestinal tract

The innate immune system uses germline-encoded pattern recognition receptors (PRRs) for the initial detection of microorganisms. PRRs recognize known microbial-specific molecular markers, such as pathogen-associated molecular patterns (PAMPs), and self-generating molecules derived from damaged cells, called damage-associated molecular patterns (DAMPs) (79). Mammals have several different types of PRRs, including TLRs. The innate immune system recognizes PAMPs derived from various microbes (80). TLRs are expressed in innate immune cells, such as dendritic cells, macrophages, and non-immune cells (81). According to their location, TLRs are roughly divided into two subfamilies: cell surface TLRs and intracellular TLRs. TLR1, TLR2, TLR4, TLR5, TLR6, and TLR10 are generally regarded as extracellular receptors. The TLR3, TLR7, TLR8, and TLR9 families are intracellular receptors located in the endosomal compartment and are responsible for recognizing viruses, bacterial-derived nucleic acids, and the host (82, 83, 84). Their signal transduction pathways also differ. The two main adapter pathways observed in TLR signaling transduction are myeloid differentiation primary response 88 (MyD88), and TIR-domain-containing adapter-inducing interferon-β pathway (81, 85).

TLRs are present in the majority of intestinal epithelial cells. TLRs change the dynamics of intestinal epithelial crypts by changing the proliferation and apoptosis of stem cells (86, 87, 88, 89). It has been demonstrated that TLR signaling is essential for epithelial repair following injury (90). After TLRs identify abnormal microbes, they can induce tighter intercellular connections, thus increasing the defense function of the intestinal epithelium (91). TLR-mediated signaling pathways, for example, regulate mucin secretion, contributing to local bacterial colonization (92). The TLR-mediated signaling pathway can promote the secretion of trefoil factor 3, amphiregulin, and prostaglandin E2, thereby promoting the repair of the intestinal epithelium (93, 94). The TLR signaling pathway can prevent the host from overreacting to the gut microbiota. If the function of TLRs is abnormal, the homeostasis between the intestinal microbes and the host changes, resulting in a decrease in pathogen clearance efficiency, causing inflammation or tumors (65, 66, 67, 68, 69, 70, 71, 72, 73, 74, 75, 76, 77, 78, 79, 80, 81, 82, 83, 84, 85, 86, 87, 88, 89, 90, 91, 92, 93, 94, 95, 96, 97).

## Abnormal changes in TLRs, especially TLR4, are related to PCOS phenotype

Research has shown that eicosapentaenoic acid can improve PCOS in rats by regulating the sterol regulatory element-binding protein 1 (SREBP-1)/TLR4 pathway (98, 99). During hyperandrogenism, inflammation induced by lipopolysaccharide (LPS) combined with TLR4 leads to the activation of inflammatory corpuscles of NLRP3, which, in turn, affects follicular dysfunction and ovarian fibrosis in rats (100). In PCOS patients, abnormal changes in TLR4 are related to abnormal metabolism, insulin resistance, and low follicular quality. Compared with thin PCOS patients, obese PCOS patients have increased LPS and TLR4 protein levels in the circulation (101). Some scholars have studied the relationship between TLR2 and TLR4 coding gene polymorphisms, sex hormones, and metabolism in PCOS patients. It was found that TLR2 s450 mutant alleles were associated with increased triglycerides, fasting insulin levels, and insulin resistance in obese women. This is related to the increase in androstenedione levels. Homozygotes of the gene k469e and K469 in intercellular adhesion molecule 1 (ICAM1) alleles only exist in obese patients with PCOS. K469 alleles were also associated with increased body mass index and diastolic blood pressure (102). The abnormal expression of TLRs in PCOS patients may reduce oocyte quality and fertility (103).

## Toll-like receptors/NF-κB signaling pathway plays an important role in PCOS

Two bioinformatics analyses found that the expression levels of TLR2, TLR8, and CD14 in PCOS patients were significantly increased; TLRs, nod-like receptors, and NOTCH signaling pathway were obviously enriched in granulosa cells (104, 105). Ren *et al.* found that TLR4 signaling pathway was enhanced in PCOS-like rats, suggesting that TLR4 signaling pathway may be related to PCOS (106). Wang *et al.* found that TLR4 was also involved in letrozole-induced changes in intestinal microbiota in PCOS rats (107). Tenalin-c (TNC) knockdown ameliorated oxidative stress and inflammation in PCOS rats and cell models by inhibiting the TLR4/NF-κB signaling pathway and inhibiting IR pathway (108). Midbrain astrocyte-derived neurotrophic factor (MANF) ameliorated DHEA-induced ovarian dysfunction and fibrosis by inhibiting TLR4-NF-κB-NLRP3 pathway (109). By down-regulating the expression of forkhead box transcription factor O1 (FoxO1), TLR4/NF-κB/NLRP3 pathway can be inhibited to alleviate PCOS and reduce inflammation and immune response (110). Cryptotanshinone improves the symptoms of PCOS rats by downregulating the HMGB1/TLR4/NF-κB pathway (111). Quercetin can inhibit the expression of TLRs in the ovaries of PCOS rats, which may improve the ovarian inflammatory microenvironment by downregulating the TLR/NF-κB signaling pathway (112). Androgen-induced TLR4/IRF-7/NF-κB signaling pathway can promote the synthesis of endometrial cytokines and increase the incidence of endometrial inflammation in patients with PCOS, while metformin can inhibit this signaling pathway (113). Bailing capsule has a therapeutic effect on PCOS through the gut-derived LPS-TLR4 inflammatory pathway (114). Liu *et al.* showed that ghrelin alleviates inflammation in PCOS mice through TLR4-NF-κB signaling pathway (115).

Toll receptors or related pathways play an important role in the process of androgen origin, transformation, and synthesis. *In vitro* experiments showed that 5-dihydrotestosterone (DHT) enhanced androgen receptor, TLR4, IRF-7, and p-NFκB p65 protein expression, along with increased interferon α (IFNα) and IFNɣ abundance (113). Studies have shown that pioglitazone, a chemotherapeutic drug reduces oxidative stress and inflammation and improves serum testosterone levels through TLR4/MyD88/NF-κB signaling pathway (116). LPS can activate the NF-κB pathway by binding to TLR4, upregulate the expression of key regulatory genes involved in androgen biosynthesis pathway Cyp17a1, Cyp11a1, Hsd3b, and Hmgcr, and promote the increase of androgen levels (117). Wuzi-Yanzong formula can inhibit the TLR/MyD88/NF-κB pathway, repair the blood-testis barrier by regulating testosterone and androgen receptor, and affect the serum testosterone level (118).

Studies on TLRs, TLR signaling pathways, and PCOS mainly focus on their effects on ovarian, systemic glucose and lipid metabolism, sex hormone changes, and endometrium. No reports have been published on gut microbiota. TLRs and TLR signaling pathways are known to play important roles in intestinal microecology and intestinal immune regulation. We hypothesize that the changes in TLR or TLR signaling pathways in the intestinal tract may be related to the development of hyperandrogenism in PCOS patients, and may also play a role in the influence of hyperandrogenism on PCOS phenotype, which deserves further investigation.

## Lower genital tract microbiota

The female lower reproductive tract includes the cervix and vagina, in which trillions of bacteria are colonized. Even though the lower reproductive tract is the simplest microecosystem of the human body, there is still insufficient understanding of it. The human vagina is an often-overlooked organ that is not merely a passageway for vaginal discharge, menses, sperm, and neonates, but can profoundly affect the health of generations. In the past 10 years, metagenomic analysis has enabled researchers to outline the vaginal microbial ecosystem. The dominant strains detected in the vagina of most healthy women were *L. crispatus, L. gasseri, L. iners*, and *L. jensenii* (119). Of note, a fifth non-Lactobacillus-dominated microbial community has also been reported in healthy women and is characterized by strictly anaerobic bacteria, such as *Atopobium*, *Dialister*, *Gardnerella*, *Megasphaera*, *Prevotella*, and *Peptoniphilus* (120). *Lactobacillus* species play a foundational role in maintaining homeostasis in the female reproductive tract. *Lactobacilli* are the most prevalent and often numerically dominant microorganisms, and are relevant as a barrier to reproductive tract infection. *Lactobacillus* produces lactic acid and hydrogen peroxide, and maintains an acidic environment with a pH of 4.0, which is not conducive to the growth of catalase-negative bacteria. *Lactobacillus* deficiency in the cervicovaginal region is associated with higher levels of genital proinflammatory cytokines, increased activation of genital antigen-presenting cells through the LPS induction pathway, and increased genital CD4+T cell counts, which activate the local immune response (121, 122). Estrogen levels play a key role in vaginal bacterial colonization. The increase in estrogen causes further thickening of the vaginal epithelial cells. Estrogen secretion increases during puberty, and the vaginal microbiota becomes similar to that of the vaginal microbiota of adult females (123). The drastic decrease in estrogen levels during menopause leads to further changes in vaginal microbiota composition (124).

## Microbiota regulate inflammation and immune response in the lower reproductive tract through the TLR pathway

The TLR is a key signaling pathway by which the cervicovaginal microbiota modulates immunity and inflammation in the female lower reproductive tract (121, 122). The cervicovaginal microbiota recognizes bacterial LPS through TLR4, activates the NF-κB pathway, triggers inflammation, recruits lymphocytes, and increases the risk of infection (121). Cytokines induced by TLR activation are important in the regulation of antimicrobial immunity and inflammatory responses in the female reproductive tract (123, 124). A cross-sectional and longitudinal study on cervicovaginal microecology in South African women found that the high diversity of cervicovaginal microbiota is closely related to the concentration of genital proinflammatory cytokines. Transcriptional profile analysis showed that genital antigen-presenting cells sense gram-negative bacterial products *in situ* via TLR4 signaling, which recruits lymphocytes by activating the NF-κB signaling pathway and producing chemokines, leading to genital inflammation (121). Pathway enrichment analysis suggested that TLRs, cytokine production, and other components of the innate immune response were associated with the community state type composed of *L. crispatus* and *L. iners* in Kenyan women (125). *Lactobacillus* can downregulate TLRs to inhibit cervicovaginal inflammation (126, 127, 128). Studies have shown that activation of TLR2 and TLR4 by low molecular weight hyaluronic acid in the epidermis can induce the secretion of antimicrobial peptide β-defensin 2, which is independent of its classical receptor CD44 and depends on the Akt/phosphatidylinositol 3 kinase pathway (129). SCFAs (short-chain fatty acids) produced by microbiota also have a significant impact on female reproductive tract immunity and inflammatory responses (130). Paria pointed out that SCFA, especially when combined with specific TLRs, can induce an inflammatory environment in the female lower reproductive tract (131). The relative abundance of *Streptococcus agalactiae* in aerobic vaginitis was higher, and more than half of the aerobic vaginitis samples contained anaerobes Gardnerella vaginalis and *Prevotella bivia*. However, despite the increased abundance of these pathogens, the aerobic vaginitis samples showed only slight stimulation of TLR4 and reduction in the activity of TLR2 and TLR6. The study pointed out that the enrichment in vaginal pathogens was the result of inflammation and/or hormonal changes in the vagina, but not the main cause of vaginitis (132). Therefore, vaginal epithelial cells are not only a simple physical barrier, but also play a role in innate host defense.

## PCOS and androgens cause changes in the lower genital tract microbiota

A number of studies have revealed a correlation between changes in the gut microbiota and PCOS. However, few studies have explored the relationship between the lower genital tract microbiota and PCOS. In 1996, Singh *et al.* found that the vaginal microbiota in PCOS rats showed some changes: the vaginal microbiota of normal rats showed periodic fluctuations, while PCOS rats did not show periodic fluctuations; in PCOS rats with persistent estrus, the loss of periodic ovarian activity led to an increase in bacterial colonization in the vaginal-cervical mucosa; leukocyte inflow resulted in a significant decrease in anaerobic bacteria and aerobic bacteria (133).

The specific mechanisms and pathways by which sex hormones affect white blood cell concentration and vaginal microbiota abundance remain unclear. Recently, a research group collected 97 pairs of vaginal and cervical canal samples from 97 women with an average age of 30 years, and 47 of them were diagnosed with PCOS. They found that there was no statistically significant difference between the cervical microbiota and vaginal microbiota of the same individual, while the content of *Lactobacillus* in the vagina and cervical microbiome of patients with PCOS was lower. Among the lower reproductive tract microbes of these patients, *Gardnerella vaginalis, Prevotella, Mycoplasma hominis*, and other non-lactic acid bacteria groups were more abundant (21).

A similar study also found that the vaginal microbiota structure of women with PCOS was significantly different from that of normal women. The vaginal microbiota in the PCOS group was more diverse than that in the control group. The relative abundance of *L. crispatus* was significantly lower than that of the control group. In the PCOS group, the relative abundance of *L. crispatus* was significantly lower, but the relative abundance of *Mycoplasma* and *Prevotella* was significantly higher. The study pointed out that *Mycoplasma* may be a potential biomarker for PCOS screening, and subgroup analysis also showed that these correlations did not change in women with the same BMI level and vaginal cleanliness level (134). In women with PCOS, the composition of lactic acid bacteria is significantly reduced, and the risk of bacterial vaginosis (BV) and vulvovaginal candidiasis is higher (18). Compared with healthy women, women with PCOS show an abnormal vaginal microbiome and an increased prevalence of BV (135).

An animal experiment found that flaxseed oil intervention significantly modulated the composition of gut microbiota and vaginal microbiota by increasing the abundances of *Allobaculum*, *Lactobacillus*, *Butyrivibrio*, *Desulfovibrio*, *Bifidobacterium*, *Faecalibacterium*, and *Parabacteroides,* as well as decreasing the abundances of *Actinoteria*, *Bacteroides*, *Proteobacteria*, and *Streptococcus,* and the ratio of *Firmicutes*/*Bacteroidetes*. Correlation analysis showed that there was a close relationship between sex steroid hormones, inflammation, and intestinal and vaginal microbiota. This suggests that flaxseed oil can improve PCOS in rats through the sex hormone–microbe–inflammatory axis (136).

At the same time, it was found that under the premise of feeding the same diet, PCOS rats with high androgen induced by letrozole had lower abundance and diversity of vaginal microbiota than normal rats, and the types of microbiota were also significantly different (136). Gabrielle compared the vaginal microbiota of cisgender women who were born female but used androgens to become transgender men. They found that *Lactobacillus* was not the main microbiota in the vagina of transgender men, and a significantly increased relative abundance of 30 species, as well as higher α diversity; intravaginal use of estrogen can reduce this difference and increase *Lactobacillus* colonization (137).

## The vagina is not only a target organ for androgens, but also an organ for androgen synthesis

At present, the influence of androgens on vaginal structure and function, and their mechanisms have not been fully elucidated. Studies have shown that androgens can affect vaginal androgen receptors, and adding androgens can improve the vaginal environment and sexual experience (34). Witherby found that androgens can improve the symptoms of vaginal atrophy without aromatizing to estradiol (138). It has been suggested that androgens have other effects on the vagina besides being a precursor of estrogen (139). The latest research points out that the vagina is not only the target organ of androgens, but also the organ for the synthesis of androgens (140). In 1995, Labrie reported for the first time the discovery of sex hormone synthesis enzymes in the vagina of rats, which laid a foundation for subsequent studies on vaginal endocrine function (141). Some studies have reported the mRNA expression of steroid enzymes in the distal vaginal tissues of perimenopausal and postmenopausal women. They found that compared with ovarian tissues, the expression of steroidogenic acute regulatory proteins and other enzymes involved in the first reactions was extremely low, but the genes related to androgen synthesis were higher, and the three subtypes of 5α-reductases (especially SRD5a2) were significantly higher than those in the ovaries. It has been reported that enzymes related to sex hormones in the vagina are more related to androgen synthesis (142).

## Concluding remarks

**Figure 1:**
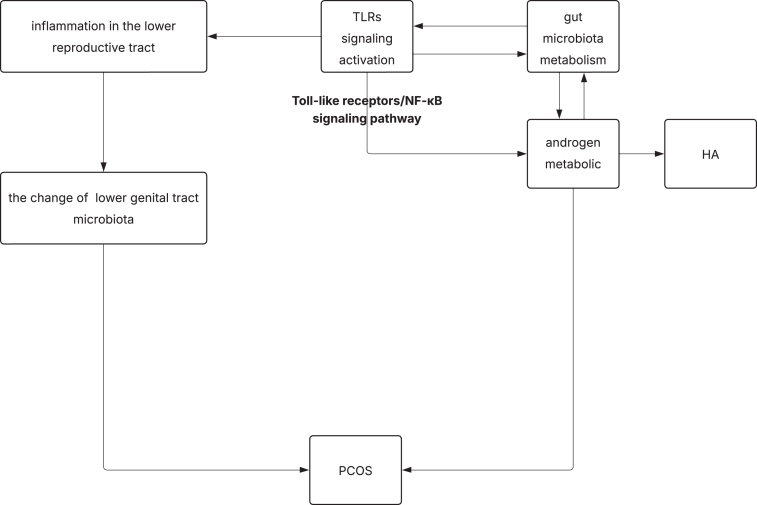
The summary of the role of microbiota and TLRs in PCOS.

We summarized the microecological characteristics of the intestinal tract and lower reproductive tract in PCOS patients and animal models, and the roles of androgens and TLRs among them. A summary of the above is presented in Fig. 1. The microbiota is not only a physical barrier, but also has physiological functions, such as regulating metabolism, inflammation, and immunity. The change in gut microbiota in PCOS is causally related to the phenotype, but the underlying mechanism is unclear. However, there are few studies on the lower reproductive tract microbiota in PCOS, and how this microbiota regulates PCOS needs further investigation. For future research, we need to clarify: i) the specific role of specific microbes in the intestinal and lower reproductive tract in PCOS; ii) the mechanism by which the microbiome affects the pathogenesis, progression, and phenotype of PCOS; iii) the dynamic changes in the intestinal and lower reproductive tract microbiota during the progression of PCOS, and its relationship with the dynamic changes in metabolism and sex hormones; and iv) the possible connection between intestinal and lower reproductive tract microbiota in PCOS patients.

In the future, accurate regulation of intestinal and lower reproductive tract microecology may improve the phenotype of patients with PCOS. Thus, FMT may be an innovative treatment option.

## Declaration of interest

All authors have completed the ICMJE uniform disclosure form. The authors declare that they have no known competing financial interests or personal relationships that could have appeared to influence the work reported in this paper. No other author has reported a potential conflict of interest relevant to this article.

## Funding

This study was supported by the Middle/Young aged Teachers' Research Ability Improvement Project of Guangxi Higher Education (grant no. 2024KY0099), Guangxi Natural Science Foundation (grant no. 2025GXNSFAA069050), and the Young Medical Reserve Talent Training Program of Guangxi province.

## Author contribution statement

Xueming Huang contributed to conceptualization, investigation, and writing of the original draft. Liuyan Wu contributed to investigation and writing of the original draft. Yunxiang Zhang contributed to investigation and writing of the original draft. Xiaowen Lai contributed to investigation and writing of the original draft. Dan Sun contributed to resources and writing (review and editing).
